# The tryptophan-aspartate (WD) repeat domain of bovine Coronin-1A promotes mycobacterial survival by inhibiting calcium signaling-mediated phagosome-lysosome fusion

**DOI:** 10.1186/s13567-025-01471-6

**Published:** 2025-02-07

**Authors:** Jing Yang, Zhunan Li, Aicong Li, Yayi Liu, Xinyan Zhang, Yong Zhang, Yuanpeng Gao

**Affiliations:** 1 Key Laboratory of Livestock Biology, Northwest A&F University, Yangling, 712100 Shaanxi China; 2 College of Veterinary Medicine, Northwest A&F University, Yangling, 712100 Shaanxi China

**Keywords:** *Mycobacterium tuberculosis*, bovine Coronin-1A, WD repeat domain, phagosome-lysosome fusion

## Abstract

Bovine tuberculosis is a chronic consumptive zoonosis, causing significant economic losses and critical public health risks. Coronin-1A is a host cytoskeleton-associated protein that is crucial for understanding the inhibition of phagosome-lysosome fusion by *Mycobacterium tuberculosis* (*M.tb*) to evade host innate immune clearance. However, the involvement of bovine Coronin-1A (bCoronin-1A) in *M.tb* infection and whether it can be manipulated so as to enhance host resistance against bovine tuberculosis remains to be seen. Here, we explored the role of bCoronin-1A in phagosome-lysosome fusion in *M.tb*-infected macrophages. We found that bCoronin-1A was upregulated at both the transcriptional and protein levels following *M.tb* infection of embryonic bovine lung (EBL) cells. Notably, bCoronin-1A was recruited to *M.tb*-containing phagosomes where it hindered phagosome-lysosome fusion, leading to increased intracellular mycobacterial survival. Further investigation revealed that mycobacterial lipoamide dehydrogenase C (LpdC) interacted with a single tryptophan-aspartate (WD) unit within the WD repeat domain of bCoronin-1A to sequester it on the phagosomes. The WD repeat domain mediated a decrease in intracellular calcium levels, which reduced levels of calmodulin-dependent kinase II (CaMKII) and its activated forms, thereby inhibiting lysosomal delivery. Overall, our findings revealed that bCoronin-1A had a critical impact on mycobacterial survival in macrophages by inhibiting calcium-mediated phagosome-lysosome fusion. This suggests that targeting bCoronin-1A as a key factor influencing mycobacterial survival may be an effective breeding strategy to develop tuberculosis-resistant dairy cows.

**Supplementary Information:**

The online version contains supplementary material available at 10.1186/s13567-025-01471-6.

## Introduction

Bovine tuberculosis is a chronic consumptive zoonosis, causing substantial global economic losses to the dairy cow industry and is a serious public health threat [1, 2]. As one of the most successful pathogens known, *Mycobacterium tuberculosis* (*M.tb*) has evolved various mechanisms to evade the host immune response. Normally, *M.tb* is internalized by macrophages and then transported to lysosomes for clearance, but strategies employed by mycobacteria such as blocking phagosomal maturation and inhibiting phagosome-lysosome fusion can block lysosomal delivery [3, 4].

The inhibition of phagolysosomal fusion by *M.tb* relies on the expression of pathogen effectors and the manipulation of host molecules [4]. For instance, during *M.tb* infection, the pathogen induces cytokine-inducible SH2-containing protein (CISH) to target the vacuolar H^+^-ATPase (V-ATPase) catalytic subunit A for ubiquitination and proteasome degradation, thereby hindering phagosomal acidification and maturation [5]. Protein kinase G (PKnG) inhibits phagocytic maturation by interfering with the host Rab7l1 signaling pathway [6]. Lipoamide dehydrogenase C (LpdC) sequesters host Coronin-1A on the phagosomes containing Bacille Calmette-Guérin (BCG), thus preventing phagolysosomal fusion during phagocytosis [7].

Mammalian Coronin-1A, also known as P57 or TACO (tryptophan/aspartate-containing coat protein), belongs to the conserved coronin family and is widely expressed in eukaryotic cells [8, 9]. Studies have highlighted its role in actin cytoskeleton dynamics, pathogen survival, autoimmune response generation, and neurobehavioral processes [10–15]. In phagocytes, live pathogenic mycobacteria recruit and retain Coronin-1A trimers on the phagosomes, aiding in mycobacterial survival by activating the Ca^2+^/calcineurin pathway to prevent lysosomal delivery [16, 17]. *M.tb* infection results in a Coronin-1A-dependent increase in cyclic adenosine monophosphate (cAMP) levels near the phagosomes, enhancing cofilin 1 to depolymerize filamentous actin (F-actin) surrounding the mycobacterial phagosomes, thereby delaying phagosome maturation [18].

Although many studies have highlighted the role of Coronin-1A in blocking lysosomal delivery of *M.tb*, evidence demonstrating the role of Coronin-1A in cows is currently lacking. In this study, we elucidated the function of bCoronin-1A after *M.tb* infection in macrophages. We further show that the WD repeat domain of bCoronin-1A interacted with LpdC to retain bCoronin-1A on phagosomes and induce a decrease in intracellular Ca^2+^ that inhibited *M.tb* from being transported to lysosomes, ultimately promoting its intracellular survival. Our results indicate that bCoronin-1A is an important factor that increases *M.tb* survival in macrophages by regulating intracellular calcium.

## Materials and methods

### Cell culture and transfection

Embryonic bovine lung (EBL), mouse RAW264.7 and human 293 T cells were cultured in high-glucose Dulbecco modified Eagle medium (DMEM) (Pricella, Wuhan, China) supplemented with 10% fetal bovine serum (FBS) (ExCell, Jiangsu, China) at 37 °C in 5% CO_2_. Polyethylenimine (PEI) transfection reagent (Polysciences Inc, Shanghai, China) was used according to the manufacturer’s instructions to introduce plasmids into cultured cells. Using RAW264.7 cells with a confluency of 60–80% in a 6-well plate as an example, the culture medium was replaced with 2 mL of serum-free DMEM 1–2 h before transfection. Then, 2 μg of plasmid DNA was added to an Eppendorf tube containing 200 μL of DMEM (10% of the total volume) and mixed thoroughly. Subsequently, 6 μL of PEI transfection reagent was introduced, maintaining a PEI-DNA ratio of 3:1. The mixture was allowed to stand for 15 min. The PEI-DNA complex was added dropwise to the cell culture plate and incubated at 37 °C for 18–24 h. After incubation, the medium was replaced with DMEM supplemented with 10% FBS and cells were further incubated for different periods.

### Mycobacterial culture and infection

The H37Ra (25177, ATCC) and BCG strains of *M. tuberculosis* were cultured in Middlebrook 7H9 broth or on 7H10 agar (Sigma-Aldrich, USA) plates supplemented with 10% oleic albumin dextrose catalase (Solarbio, Beijing, China) and 0.5% glycerol. The required number of bacteria was centrifuged at 4500 × *g* for 5 min, washed with phosphate-buffered saline (PBS), and resuspended in DMEM containing 10% FBS for subsequent cell infection. RAW264.7 cells were primarily infected at a multiplicity of infection (MOI) of 10, and for immunofluorescence (IF) assay and measurement of H37Ra binding to macrophages, they were infected with an MOI of 20. EBL cells were infected at an MOI of 20. After infection, the cells were incubated at 37 °C in 5% CO_2_ for 4 h and washed three times with PBS to remove non-internalized mycobacteria. Subsequently, the medium was replaced with fresh culture medium, and cells were further incubated for different periods.

### Cell viability assay

Cell viability was determined using a cell-counting 8 (CCK8) kit (Beyotime, Shanghai, China). RAW264.7 cells were plated at a density of 1 × 10^4^ cells/well in a 96-well plate. After transfection and following infection with *M.tb* at an MOI of 10, 10 μL of CCK8 reagent was added to each well and incubation was continued for 1–4 h. Absorbance was measured at 450 nm with a microplate reader (Thermo Fisher Scientific, USA).

### Colony-forming unit (CFU) assay

RAW264.7 cells (5 × 10^5^) were grown in 12-well plates, infected with H37Ra/BCG at an MOI of 10 for 4 h, and washed three times with PBS to remove extracellular mycobacteria. Infected cells were cultured for the indicated time and then lysed in PBS containing 0.1% TritonX-100 for 5 min at room temperature. The lysates were centrifuged, resuspended in PBS, serially diluted, plated in triplicate on Middlebrook 7H10 agar plates, and incubated at 37 ℃ for 1–2 weeks before colony counting.

### Immunofluorescence (IF) assay

RAW264.7 cells (1 × 10^5^) infected with H37Ra/BCG at an MOI of 20 for 4 h, were grown overnight at 37 ℃ on Teflon-coated glass slides with 24-well chambers (NEST, Wuxi, China). After fixation with fixative immunostaining (Beyotime, Shanghai, China) for 20 min and permeabilization/blocking for 1 h with immunostaining blocking buffer at room temperature (Beyotime, Shanghai, China), cells were incubated overnight at 4 ℃ with primary antibodies anti-LAMP1 (Abcam, ab208943, 1:100) and anti-β-tubulin (TransGen Biotech, HC101-01, 1:500). After washing three times with TBSTx (20 mM Tris, 140 mM NaCl, 0.1% TritonX-100, pH7.6), Cy3-labeled goat anti-rabbit IgG (H + L) (Beyotime, A0516, 1:1000) and FITC-labeled goat anti-mouse IgG (H + L) (Beyotime, A0568, 1:500) were added and the slides were incubated in the dark for 1 h at room temperature. The slides were washed and stained with DAPI (Beyotime, C1002, 1:4000), mounted using antifade mounting medium (Beyotime, Shanghai, China), imaged with a confocal microscope (Leica, Germany) and analyzed using the corresponding software.

### Measurement of H37Ra binding to macrophages

The effect of bCoronin-1A on H37Ra binding to the RAW264.7 was evaluated according to a previously described method [19]. RAW264.7 monolayers on Teflon-coated glass slides with 24-well chambers were washed twice with binding medium (138 mM NaCl, 8.1 mM Na_2_HPO_4_, 1.5 mM KH_2_PO_4_, 2.7 mM KCl, 0.6 mM CaCl_2_, 1 mM MgCl_2_, and 5.5 mM D-glucose) and acclimatized for 10 min at 37 °C in 5% CO_2_. Before infection, H37Ra was stained with 1 mM FITC (MedChemExpress, Shanghai, China) at 37 °C for 1 h, and then washed twice with PBS. Host cells were infected with FITC-labeled H37Ra at an MOI of 20 for 1 h, non-adherent bacteria were washed off, and infection was continued for 3 h at 37 ℃. Cells were washed three times, fixed, and stained with DAPI (Beyotime, C1002, 1:4000) for 10 min. The number of cells binding to at least one mycobacterium in five fields in each well was counted under the microscope. In a separate experiment to measure the FITC fluorescence intensity, RAW264.7 cells were incubated with FITC-labeled H37Ra for 4 h, washed with PBS, and added to a black 96-well plate at 100 μL/well. Each sample was run with three replicates. FITC fluorescence was measured with excitation at 488 nm and emission at 512 nm using a microplate reader.

### Isolation of mycobacteria-containing phagosomes

The phagosome isolation was performed as described previously [20]. RAW264.7 cells infected with H37Ra at an MOI of 10 were washed and harvested by centrifugation. Pellets were resuspended in 1.5 mL homogenization buffer, then lysed using a Dounce homogenizer (30–40 times) and passed through a 1 mL syringe with 27-gauge needle (20 times). The sample was centrifuged at 800 × *g* for 5 min at 4 ℃ to remove nuclei and intact cells. The supernatants were layered onto sucrose gradients (2 mL 50% sucrose, 4 mL 37% sucrose and 4 mL 25% sucrose) in a centrifuge tube and centrifuged in an SW41 rotor at 100 000 × *g* for 60 min at 4 °C. Mycobacterial phagosomes located between 50 and 37% sucrose were collected by a 27-gauge needle. The phagosome suspension was layered onto 2 mL 50% sucrose and centrifuged again in an SW60 rotor at 100 000 × *g* for 30 min at 4 °C. The mycobacteria-containing phagosomes were observed as a band above the 50% sucrose layer and collected as described above.

### Lysosome isolation

RAW264.7 cells infected with FITC-labeled H37Ra at an MOI of 10 were collected for lysosome isolation using a lysosome isolation kit (BestBio, Shanghai, China) according to the manufacturer’s instructions. The fluorescent intensity of H37Ra was measured using a CytoFLEX flow cytometer (Beckman, China) with excitation at 488 nm and the results analyzed with Flowjo_v10.8.1 software. Gate settings of FACS were established using lysosomes extracted from cells transfected with p3 × Flag.

### Measurement of lysosomal pH

The LysoSensor™ yellow/blue DND-160 kit (Yeasen, Shanghai, China) was used according to the manufacturer’s protocol. Briefly, 1 × 10^5^ RAW264.7 cells were seeded on coverslips in a 24-well plate and infected with H37Ra at an MOI of 10 for 4 h, then washed three times with PBS, and loaded with 1 μM LysoSensor in culture medium for 1 h at 37 ℃. The cells were then washed three times with PBS and immediately observed and imaged by fluorescence microscopy (Olympus, Japan).

For measurements of fluorescence intensity, cells were washed and harvested with PBS, then added to a black 96-well plate at 100 μL/well with three replicates per sample. Fluorescence values were measured using a microplate reader with fluorescence excitation at 329 nm and emission at 440 nm (blue), and fluorescence excitation at 384 nm and emission at 540 nm (yellow).

### RNA isolation, cDNA reverse transcription, and quantitative real-time polymerase chain reaction (qRT-PCR)

Total RNA was extracted using the TRIzol reagent (TransGen Biotech, Bejing, China), RNA was reverse transcribed using a HiScript II Q RT SuperMix for qPCR (+ gDNA wiper) (Vazyme, Nanjing, China). The qPCR analysis was performed on a CFX96™ real-time PCR system (Bio-Rad, USA) with ChamQ Universal SYBR qPCR Master Mix (Vazyme, Nanjing, China). Bovine *GAPDH* was used to normalize the relative expression of mRNA and the results were analyzed using the 2^−ΔΔCt^ method. Primer sequences are as follows:

*bCoro1a* (forward, 5′-CCCCTCCTCATCTCCCTCAA-3′, reverse, 5′-GGACTGTCTCCTCCAGCCTA-3′),

*mMfsd8* (forward, 5′-GCCAACTGCCTATATGCGTATGTC-3′, reverse, 5′-CTGCTCCAAATCCCACCAATCC-3′),

*mCtsd* (forward, 5′-ACGGAGCCAGTGTCAGAGTTAC-3′, reverse, 5′-CCACAGGTTAGAGGAGCCAGTATC-3′),

*mCtsf* (forward, 5′-GGCAACCGCTCTAACATTCCTTAC-3′, reverse, 5′-TTCACACCACAGGCTCCAGATC-3′),

*mSnap29* (forward, 5′-CCATTGACAGGCAGCAGTACC-3′, reverse, 5′-GACTCCGATCTTCTCCGATTCATAC-3′),

*bGAPDH* (forward, 5′-GAGCGAGATCCTGCCAACAT-3′; reverse, 5′-GGTTCACGCCCATCACAAAC-3′).

*mGAPDH* (forward, 5′-AGGTCGGTGTGAACGGATTTG-3′; reverse, 5′-TGTAGACCATGTAGTTGAGGTCA-3′).

### Western blot

Proteins were extracted using RIPA lysis buffer (Beyotime, Shanghai, China), separated on a 10% SDS-PAGE gel, and electroblotted onto polyvinylidene fluoride membranes (Millipore, Ireland). The membranes were blocked with 5% nonfat dry milk in TBST (20 mM Tris, 150 mM NaCl, 0.1% Tween-20, pH 7.0) for 2 h at room temperature, and incubated with primary antibody overnight at 4 ℃. The primary antibodies included GAPDH (Proteintech, 60004-1-Ig, 1:3000), Coronin-1A (Proteintech, 17760-1-AP, 1:1000), LAMP1 (Proteintech, 67300-1-Ig, 1:1000), EEA1 (Proteintech, 68065-1-Ig, 1:1000), GFP (TransGen Biotech, HT801-01, 1:1000), FLAG (Cell Signaling Technology, 14793, 1:1000), HA (Cell Signaling Technology, 3724, 1:1000), CaMKII (Proteintech, 13730-1-AP, 1:1000), and phospho-CaMKII (Affinity, AF3493, 1:1000). After incubation with primary antibodies overnight at 4 °C, the membranes were washed and incubated with the corresponding second antibodies, HRP-labeled goat anti-rabbit IgG (H + L) (Beyotime, A0208, 1:2000) or HRP-labeled goat anti-mouse IgG (H + L) (Beyotime, A0216, 1:2000) respectively for 1 h at room temperature. The bands were visualized by enhanced chemiluminescence (Advansta, USA) and imaged using a photodocumentation system.

### Co-immunoprecipitation (Co-IP)

After co-transfection of 293 T cells for 48 h with plasmids encoding LpdC and bCoronin-1A, bCoronin-1A’s truncated forms and other members of the coronin family, the cells were lysed with IP lysis buffer (20 mM Tris–HCl pH7.5, 150 mM NaCl, 1 mM Na_2_EDTA, 1% NP40, 1 mM PMSF) and then centrifuged at 12 000 × *g* for 15 min at 4 °C. The supernatants were incubated with anti-FLAG M2 beads (Sigma, USA) or anti-HA Affinity beads (Smart-Lifesciences Biotechnology, Changzhou, China) at 4 °C overnight on a rotator to form the immune-complex. The beads were washed five times with cold IP lysis buffer and boiled with 70 μL 1 × SDS loading buffer for 10 min at 100 °C. The samples were analyzed by western blot.

### Native and SDS-PAGE

Native and SDS-PAGE were used to detect the presence of oligomeric or monomeric forms of proteins under native and denatured conditions, respectively. After transfection of 293 T cells for 48 h with plasmids p3 × Flag-bCoronin-1A, p3 × Flag-ΔW and p3 × Flag-ΔC, the cells were lysed with IP lysis buffer and then centrifuged at 12 000 × *g* for 15 min at 4 °C. The supernatants were incubated with anti-FLAG M2 beads (Sigma, USA) at 4 °C overnight on a rotator to form the immune-complex. The beads were washed five times with cold IP lysis buffer and incubated with 100 μL 3 × Flag peptide (Beyotime, Shanghai, China) (150 μg/mL) in TBS (50 mM Tris–HCl, pH7.4, 150 mM NaCl) at 4 °C on a rotator for 2 h. The supernatant is the purified Flag fusion protein after centrifugation at 6000 × *g* for 30 s at 4 °C. The protein samples were boiled with 5 × SDS loading buffer for 10 min at 100 °C before running on SDS-PAGE. For native PAGE, the protein samples were mixed with 5 × loading buffer without SDS. Note that neither the gel nor running buffer for native PAGE contained SDS, but the remaining procedures were identical to those for SDS-PAGE. The bands were visualized by Coomassie Blue Fast Staining Solution (Sangon Biotech, Shanghai, China) and imaged using a photodocumentation system.

### Bimolecular fluorescence complementation (BiFc)

LpdC, bCoronin-1A and its truncated forms and other members of the coronin family were inserted into BiFC vectors pBiFC-Vn173 and pBiFC-Vc155 using ClonExpress MultiS one-step cloning kit (Vazyme, Jiangsu, China), respectively. The recombinant plasmids indicated in each experiment (0.5 μg each) were co-transfected with PEI into 293 T cells growing in 24-well plates. At 48 h after transfection, the green fluorescence in living cells was observed and imaged by fluorescence microscopy.

### Calcium measurements

After infecting cells with H37Ra at an MOI of 10 for 4 h and washing them three times with PBS, 1 μM fluo-4AM (Beyotime, Shanghai, China) in PBS was loaded into cells by incubating them for 30 min at 37 ℃ in 5% CO_2_. After incubation, the cells were washed three times and then incubated with PBS containing 1% FBS for 20 min. The calcium levels were quantitated using a CytoFLEX flow cytometer (Beckman, China) with excitation at 488 nm and emission at 512 nm, and the results analyzed using Flowjo_v10.8.1 software. The gate settings for flow cytometry were established using cells transfected with p3 × Flag without loading fluo-4AM.

### Statistical analysis

All statistical analyses were performed using GraphPad Prism 5.0. Data are means of three independent experiments with the least squares standard error of the mean (SEM) and compared by paired *t*-test. Differences were considered statistically significant at *P* < 0.05.

## Results

### bCoronin-1A was upregulated after *M.tb* infection and promoted mycobacterial survival

**Figure 1:**
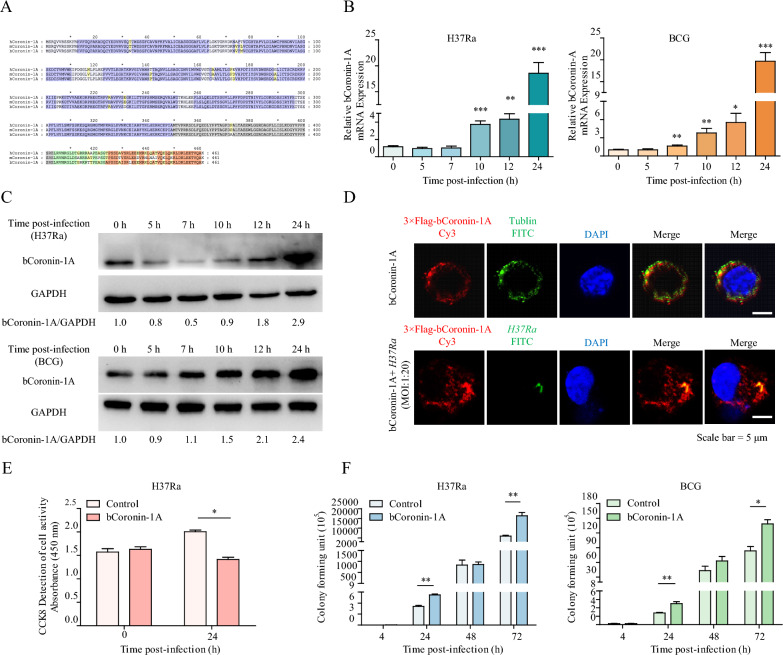
**Upregulation of bCoronin-1A promoted the intracellular survival of** ***M.tb***. **A** Protein sequence alignment of hCoronin-1A, mCoronin-1A and bCoronin-1A. The blue area indicates WD1-WD7 in the WD repeat domain, the gray area is the unique sequence, the green is the disordered sequence, and the orange part is the coiled-coil domain. **B**, **C** The mRNA and protein levels of bCoronin-1A were measured in EBL cells infected with H37Ra and BCG for 0, 5, 7, 10, 12 and 24 h by qRT-PCR and western blot (MOI = 20). **D** Immunofluorescence analysis of subcellular localization of bCoronin-1A before and after infection of RAW264.7 cells overexpressing bCoronin-1A with H37Ra (MOI = 20). The reagents used included rabbit anti-FLAG polyclonal antibody for labeling bCoronin-1A (red), mouse anti-tubulin polyclonal antibody (green) for labeling tublin and DAPI for nuclear staining (blue). **E** Detection of cell activity in RAW264.7 cells overexpressing bCoronin-1A before and after infection with H37Ra (MOI = 10). **F** CFU detection of H37Ra and BCG from infected RAW264.7 cells overexpressing bCoronin-1A at 4, 24, 48 and 72 h. Data were analyzed by *t*-test and presented as the mean ± SEM of three independent experiments. *P < 0.05, **P < 0.01, ***P < 0.001.

Through sequence alignment and structural analysis, it was determined that bCoronin-1A shows high homology with *Homo sapiens* Coronin-1A (hCoronin-1A) and *Mus musculus* Coronin-1A (mCoronin-1A), all of which contain the tryptophan-aspartate (WD) repeat region and a coiled-coil domain, characteristic structural features shared by most members of the coronin family (Figure 1A, Additional files 1A, B) [12, 21].

To determine the role of bCoronin-1A in *M.tb* infection, we first infected EBL cells with H37Ra and BCG at different time points. Following H37Ra infection, we observed a significant increase in bCoronin-1A mRNA and protein at 10 and 12 h post-infection, respectively (Figures 1B and C). In the case of BCG infection, the mRNA and protein levels of bCoronin-1A significantly increased at 7 and 10 h post-infection, respectively (Figures 1B and C). We overexpressed bCoronin-1A in RAW264.7 cells and analyzed its localization by immunofluorescence. Given the association of Coronin-1A with the cortical microtubule network, we utilized tubulin as a marker for this analysis [9]. Compared to the co-localization of bCoronin-1A and tubulin in the cortex of uninfected cells, bCoronin-1A not only exhibited cortical localization in cells infected with H37Ra for 4 h, but was also localized around the mycobacteria (Figure 1D). To assess the intracellular survival of *M.tb*, the CFU at different time points was quantified. Our results revealed that bCoronin-1A promoted mycobacterial survival while significantly reducing host cell viability (Figures 1E and F). These findings collectively indicate that bCoronin-1A was upregulated during *M.tb* infection and improved intracellular mycobacterial survival.

### bCoronin-1A facilitated *M.tb* evasion of lysosomal degradation by blocking phagosome-lysosome fusion

**Figure 2:**
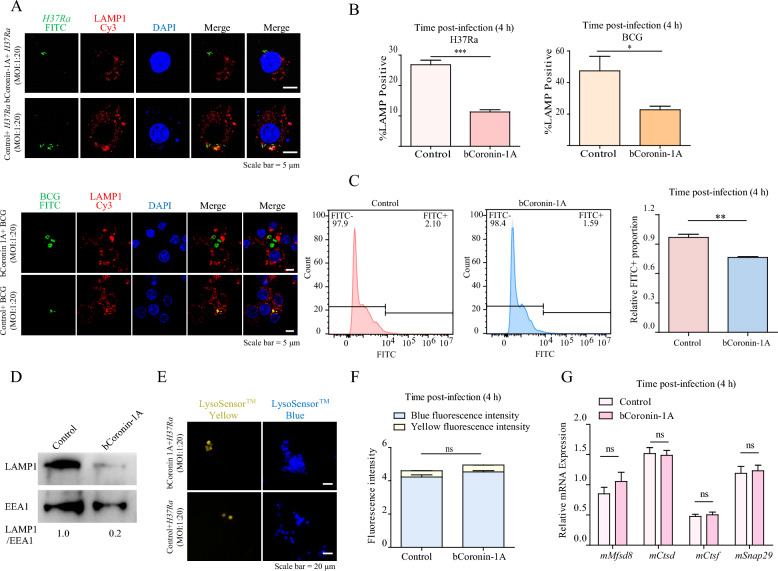
**Overexpression of bCoronin-1A facilitated** ***M.tb*** **evasion of lysosomal clearance**. **A** Immunofluorescence detection of *M.tb* localization after 4 h of infection, with FITC-labeled H37Ra/BCG (green), rabbit anti-LAMP1 monoclonal antibody for lysosomes (red), and DAPI-stained nuclei (blue) (MOI = 20). **B** Statistical comparison of percent co-localization of *M.tb* with lysosomes. **C** Flow cytometry determination of the percentage of extracted lysosomes containing FITC-labeled *M.tb* (MOI = 10)*.* **D** The degree of lysosomal fusion in extracted phagosomes was determined by western blot with mouse anti-EEA1 monoclonal antibody for phagosomes and rabbit anti-LAMP1 monoclonal antibody for lysosomes (MOI = 10). **E** Lysosomal acidification was detected using the LysoSensor™ yellow/blue probe, which produces blue fluorescence under neutral conditions and emits yellow fluorescence when the environment is acidic (MOI = 10). **F** The intensity of blue and yellow fluorescence was measured with a microplate reader. **G** The relative mRNA expression of lysosome-related genes was determined by qRT-PCR (MOI = 10). Data were analyzed by *t*-test and presented as the mean ± SEM of three independent experiments. * P < 0.05, ** P < 0.01, *** P < 0.001, ^ns^*P* > 0.05.

To further investigate the mechanism of Coronin-1A’s enhancement of the intracellular survival of *M.tb*, we first explored its impact on the binding of *M.tb* to cells by immunofluorescence. After infection with FITC-labeled H37Ra for 4 h, we observed that there was no significant difference in the adherence of *M.tb* to cells overexpressing bCoronin-1A compared to the control group (Additional file 1C–E). Lysosomal delivery is crucial for clearing *M.tb* infection. Determination of the intracellular location of *M.tb* revealed that in cells overexpressing bCoronin-1A, a significantly lower proportion of H37Ra co-localized with lysosomal-associated membrane protein-1 (LAMP1) in lysosomes compared to the control group, indicating that the majority of *M.tb* cells were retained in phagosomes instead of being delivered to lysosomes. Similar results were observed in cells infected with BCG (Figures 2A and B). Consequently, we isolated lysosomes from the cells and quantified the percentage of FITC-labeled H37Ra by flow cytometry, confirming reduced *M.tb* localization within lysosomes in the bCoronin-1A overexpressing cells (Figure 2C). Analysis of phagosomal components isolated by sucrose density gradient centrifugation showed significantly lower expression levels of LAMP1 in phagosomes from macrophages overexpressing bCoronin-1A (Figure 2D). We did not observe significant differences in lysosome acidification or the expression of lysosome-related genes between the cells overexpressing bCoronin-1A and the control group (Figures 2E–G). Collectively, these results indicate that bCoronin-1A hindered phagosome-lysosome fusion upon mycobacterial infection.

### The WD repeat domain of bCoronin-1A is critical for inhibiting the fusion of phagosome-lysosomes containing *M.tb*

**Figure 3:**
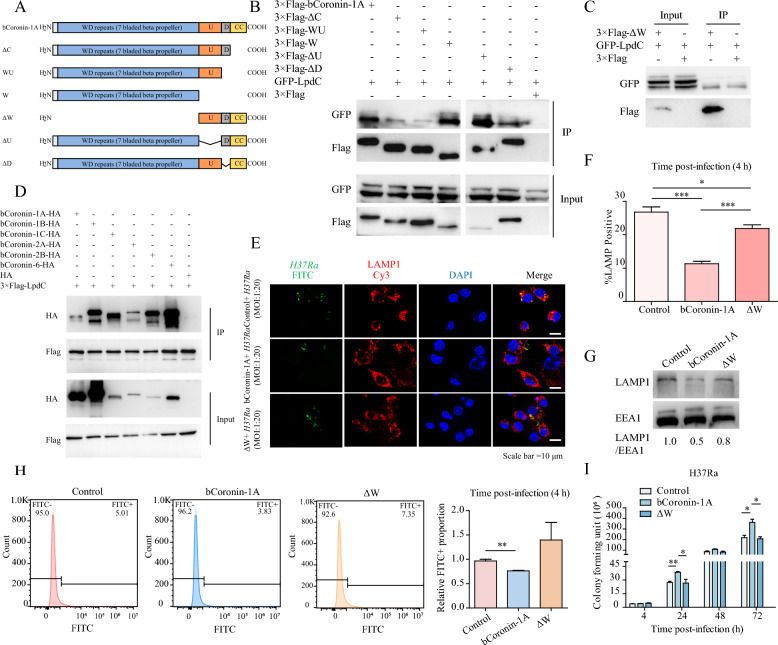
**The WD repeat domain inhibited phagolysosomal fusion after** ***M.tb*** **infection**. **A** Diagram of bCoronin-1A and its truncated forms showing the domain structure. **B**, **C** Co-IP detection of the interaction between bCoronin-1A and its truncated forms with LpdC of *M.tb*. **D** Co-IP detection of the interaction of bCoronin-1A and its family members with LpdC. **E** Immunofluorescence analysis of *M.tb* localization after 4 h of infection in cells overexpressing bCoronin-1A, ΔW, and in control cells (MOI = 20). **F** Statistical analysis of percentage of *M.tb* co-localized in lysosomes. **G** The degree of lysosomal fusion in extracted phagosomes determined by western blot (MOI = 10). **H** Flow cytometry calculation of the percentage of extracted lysosomes containing FITC-labeled *M.tb* (MOI = 10). **I** CFU assay at different times of *M.tb* infection (MOI = 10). Data were analyzed by *t*-test and presented as the mean ± SEM of three independent experiments. * P < 0.05, ** P < 0.01, *** P < 0.001.

To determine the factors influencing the retention of bCoronin-1A in phagosomes to protect *M.tb* from lysosomal clearance, we first considered *M.tb*-host interactions. Mycobacterial LpdC has been reported to be a Coronin-1A binding protein [7], and therefore, we were interested in the relationship between LpdC and bCoronin-1A. Co-transfection of the LpdC and bCoronin-1A expression vectors in 293 T cells revealed their interaction through Co-IP and BiFc assay (Figures 3A and B, Additional file 2A). To investigate which segment of bCoronin-1A mediated the interaction with LpdC, we truncated the various structural domains and sequences of bCoronin-1A (Figure 3A). Co-IP and BiFC assay results indicated that the interaction between LpdC and bCoronin-1A was abolished only when the WD repeat domain was absent (Figures 3A–C, Additional file 2A). Additionally, our investigation extended to other members of the Coronin family, such as bCoronin-1B, 1C, 2A, 2B and 6, all of which contain the WD repeat domain, and were found to interact with LpdC in vitro (Figure 3D, Additional files 2B and C). These findings suggest that the WD repeat domain serves as a critical mediator in the interaction between bCoronin-1A and LpdC.

In order to explore the role of the WD repeat domain in survival of *M.tb* upon delivery to lysosomes, we overexpressed bCoronin-1A lacking WD repeats (ΔW) in RAW264.7 cells. IF analysis demonstrated a notable increase in the co-localization of *M.tb* with lysosomes in the ΔW group compared to the bCoronin-1A group (Figures 3E and F). Elevated levels of LAMP1 expression were also detected in the isolated phagosomes of the ΔW group (Figure 3G). In the lysosomal extracts, a greater amount of FITC-labeled *M.tb* was observed in the ΔW group (Figure 3H), suggesting that the absence of WD repeat domain partially rescued the capability for lysosomal clearance of *M.tb*. Furthermore, CFU results showed a significant reduction in mycobacterial numbers in the ΔW group, indicative of lower *M.tb* survival (Figure 3I). These findings suggest that the WD repeat domain is a critical structural element in the interaction between bCoronin-1A and LpdC, thereby facilitating *M.tb* retention to evade lysosomal clearance.

### Individual WD unit of bCoronin-1A can interact with the LpdC of *M.tb*

**Figure 4:**
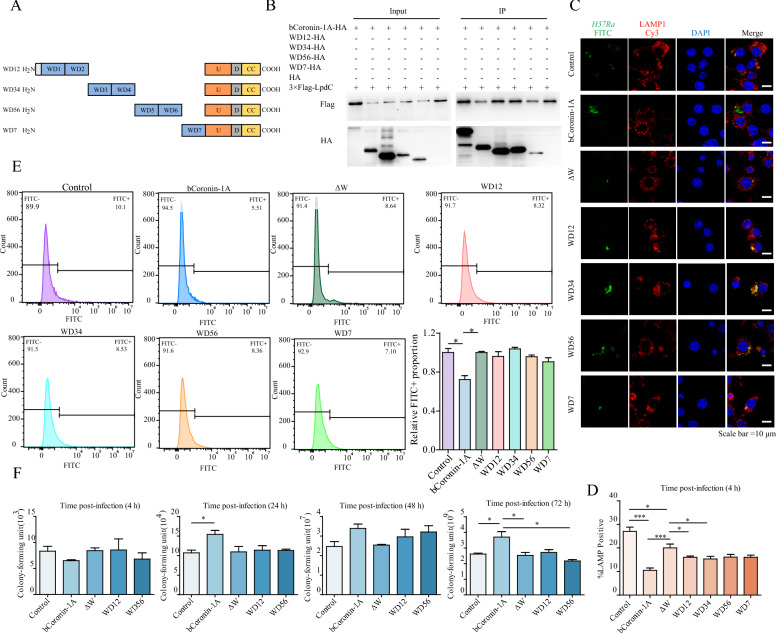
**The impact of a single WD unit on inhibiting phagolysosomal fusion after** ***M.tb*** **infection**. **A** Diagram showing truncated forms of the WD repeat domain of bCoronin-1A. **B** Co-IP of truncated WD repeat domain of bCoronin-1A interacting with LpdC of *M.tb* in vitro. **C** Immunofluorescence detection of *M.tb* localization after 4 h of infection in cells overexpressing bCoronin-1A, ΔW, truncated forms of the WD repeat domain, and in control cells (MOI = 20). **D** Statistical analysis of the percentage of *M.tb* co-localized with lysosomes. **E** Flow cytometry analysis of the percentage of extracted lysosomes containing FITC-labeled *M.tb* (MOI = 10). **F** CFU assay at different times of *M.tb* infection (MOI = 10). Data were analyzed by *t*-test and presented as the mean ± SEM of three independent experiments. * P < 0.05, *** P < 0.001.

Since the WD repeat domain is composed of seven WD units (WD1-7), which accounts for 71% of the length of the entire bCoronin-1A molecule, we truncated the WD repeat domain to determine which unit interacted with LpdC (Figure 4A). Interestingly, the Co-IP and BiFc assays revealed that LpdC could interact with a single WD unit (Figure 4B, Additional file 2D). When expressing the different truncations in macrophages and observing *M.tb* localization, it was noted that cells overexpressing WD units exhibited a minor reduction in *M.tb* co-localization with lysosomes compared to ΔW overexpression (Figures 4C and D). This trend was also observed in the isolated lysosomes (Figure 4E). Furthermore, the CFU assay showed a slight increase in intracellular proliferation of *M.tb* in the presence of one or two WD units (Figure 4F). Collectively, these results indicate that the individual WD unit contributed to the WD-mediated interaction between bCoronin-1A and LpdC, which was retained by *M.tb* to escape the lysosomal clearance process.

### The WD repeat domain mediated a decrease in intracellular Ca^2+^ and inhibited the activation of CaMKII

**Figure 5:**
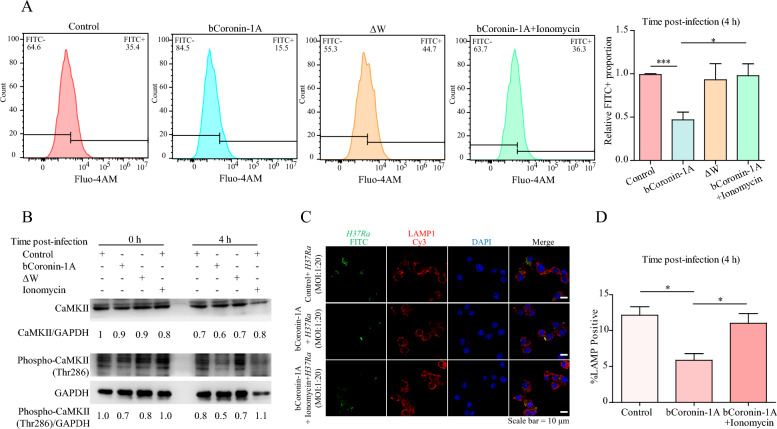
**WD repeat domain inhibited the Ca**^**2+**^**-CaM-CaMKII pathway after** ***M.tb*** **infection**. **A** Flow cytometry measurement of intracellular calcium labeled with fluo-4AM after 4 h of *M.tb* infection in cells overexpressing bCoronin-1A (loaded or not loaded with 1 μM ionomycin for 30 min at 37 ℃), ΔW and control cells (MOI = 10). **B** Western blot determination of CaMKII and phospho-CaMKII (Thr286) expression levels after 4 h of infection with *M.tb* in cells overexpressing bCoronin-1A, ΔW and control cells (MOI = 10). **C** Immunofluorescence imaging of FITC-labeled *M.tb* localization in the above cells (MOI = 20). **D** Statistical analysis of proportion of *M.tb* and lysosome co-localization. Data were analyzed by *t*-test and presented as the mean ± SEM of three independent experiments. * P < 0.05, *** P < 0.001.

Previous studies have established the crucial role of calcium in phagosome-lysosome fusion with Coronin-1A, which was known to regulate Ca^2+^ levels [17, 22]. To explore whether the effect of bCoronin-1A on *M.tb*-containing phagosome-lysosome fusion was related to intracellular Ca^2+^, we utilized the fluorescent calcium probe fluo-4AM to measure intracellular Ca^2+^ levels after 4 h of H37Ra infection. The results demonstrated a significant decrease in intracellular Ca^2+^ levels in cells overexpressing bCoronin-1A compared to the control group, whereas the Ca^2+^ levels in overexpressing ΔW cells were similar to those in the control group (Figure 5A). Subsequently, we treated cells overexpressing bCoronin-1A with 1 μM Ca^2+^ ionophore (ionomycin) for 30 min, which led to a significant elevation in intracellular Ca^2+^ levels, similar to those observed in the control group (Figure 5A). Similar results were obtained in EBL cells (Additional file 3A), suggesting that the WD repeat domain of bCoronin-1A mediated the decrease in intracellular Ca^2+^ upon *M.tb* infection.

It has been reported that *M.tb* inhibits phagolysosome fusion by suppressing the Ca^2+^-calmodulin (CaM)-CaMKII pathway [23]. To investigate the impact of WD repeat domain-mediated calcium reduction on this pathway, we measured the CaM-dependent protein kinase II (CaMKII) protein levels and its activated form in cells overexpressing bCoronin-1A and with ΔW compared with control cells. The results showed that the protein levels of CaMKII and phospho-CaMKII (Thr286) were lower in bCoronin-1A-overexpressing cells compared to control and ΔW cells, with no significant difference between the latter two groups. After ionomycin treatment for 30 min, CaMKII and phospho-CaMKII (Thr286) levels rebounded to levels similar to the control group, suggesting a recovery in protein expression (Figure 5B). We also obtained similar results in EBL cells (Additional file 3B), providing further support for the inhibition of CaMKII activation due to reduced intracellular Ca^2+^levels mediated by the WD repeat domain.

To further investigate the potential impact of WD repeat-mediated reduction of intracellular Ca^2+^on blocking the lysosomal delivery and supporting the survival of *M.tb*, we compared the localization of H37Ra in cells that either overexpressed bCoronin-1A or not, with or without ionomycin treatment. The results revealed that following ionomycin treatment, the co-localization percentage of H37Ra and lysosomes was comparable to that of the control group (Figure 5C and D). Thus, it can be concluded that the WD repeat domain was involved in reducing intracellular Ca^2+^ levels, leading to the suppression of CaMKII activation and subsequently hindering phagosome-lysosome fusion.

## Discussion

In this study, we focused on investigating the potential effects of bCoronin-1A on the *M.tb*-containing phagolysosomal fusion and evaluating whether it could be used as a potential factor in breeding for bovine tuberculosis resistance. Following *M.tb* infection, a significant upregulation of bCoronin-1A expression was observed in EBL cells. Consistent with previous research findings, the level of Coronin-1A in tuberculosis patients was significantly higher than that in healthy individuals, and the serum level of Coronin-1A was also expected to serve as a new biomarker for tuberculosis diagnosis [24, 25].

We observed a shift in the localization of bCoronin-1A from the cell cortex to the interior of macrophages, surrounding *M.tb* before and after infection, which is consistent with previous reports in which the actin cytoskeleton played a significant role in regulating phagocytosis and phagosome-lysosome fusion [9, 17, 18, 26]. F-actin has been observed to transiently accumulate around immature phagosomes in a process known as “actin-flashing” [27]. As an actin-binding protein, hCoronin-1A co-localizes with F-actin and is shed from nascent phagosomes after particle internalization, and the dissociation of mCoronin-1A on phagosomes is also necessary for phagosome-lysosome fusion [9, 28, 29]. *M.tb* causes Coronin-1A to be retained on phagosomes, which is a strategy for inhibiting phagosome maturation and enhancing mycobacterial survival. Previous studies have shown that Coronin-1A deficiency results in increased transfer of *M.tb* to lysosomes and enhanced clearance in mice and cells [17, 26]. We confirmed those findings in our bCoronin-1A overexpression cell model. The function of Coronin-1A in contributing to mycobacterial survival can be generalized to other pathogens. Both *Mycobacterium leprae* and vacuolar toxin-expressing *Helicobacter pylori* can retain Coronin-1A and hijack phagosome maturation [30, 31].

Pathogen-host interaction is the primary activity motivating our exploration of the reasons why *M.tb* retains bCoronin-1A to inhibit phagolysosomal fusion. Studies have shown that the phosphotyrosine phosphatase (PtpA) secreted by *M.tb* binds to subunit H of macrophage V-ATPase, inhibiting lysosomal acidification [32]. In *Staphylococcus aureus*, PtpA interacts with Coronin-1A and promotes pathogen survival [33]. As a virulence factor, LpdC blocks phagosome maturation by sequestering Coronin-1A in BCG vacuoles. This was first proposed by Ala-Eddine Deghmane et al. who demonstrated that the binding of LpdC to Coronin-1A required the stabilization of host cytokine cholesterol [7, 34]. Here we directly verified the interaction between LpdC and bCoronin-1A in 293 T cells, thus corroborating their findings, and further identified the WD repeat domain of bCoronin-1A as crucial for this interaction in which the WD repeats can be folded into a β-propeller structure, known to be involved in protein–protein interactions [35]. Our study revealed an important contribution of the WD repeat domain to the inhibition of phagolysosomal fusion, which may be related to its structure. Analysis of the crystal structure revealed that the sequence of Trp-Asp dipeptide WD repeats in Coronin-1A was located on the curved β-meander motif and almost completely exposed [36]. Other WD repeat-containing proteins, such as tomosyn, which inhibits membrane fusion mediated by the soluble N-ethylmaleimide-sensitive factor attachment protein receptor (SNARE) complex was also reported to have 50% of its WD sequence exposed [37, 38]. Studies have also shown that synthetic peptides derived from the Coronin-1A WD repeat domain effectively inhibited membrane fusion. These results support the hypothesis that the binding of LpdC to the Coronin-1A WD repeat domain inhibits phagolysosomal fusion [39]. The presence of a single WD unit interacting with LpdC is sufficient, which suggests that the interaction may be more complex.

In addition to the WD repeat domain, the coiled-coil domain also plays an important role in phagolysosomal fusion. The coiled-coil motif mediates trimerization of Coronin-1A, which is crucial for the activation of calcineurin and the protection of *M.tb* from lysosomal delivery [16, 40]. Serine phosphorylation of Coronin-1A by protein kinase C (PKC) catalyzes the conversion of trimers to monomers, and also induces phosphatidylinositol-3-kinase activity, thereby shifting the mode of internalization from receptor-mediated phagocytosis to macropinocytosis, causing macrophages to acquire the ability to rapidly clear pathogens [41, 42]. In the present study, we confirm that the coiled-coil domain of bCoronin-1A can cause the trimerization of Coronin-1A and block the delivery of *M.tb* to the lysosome (Additional file 4).

Calcium signaling plays important roles in actin rearrangement, formation of the reduced form of nicotinamide adenine dinucleotide phosphate (NADPH), and PKC activation during phagocytosis [22]. The Ca^2+^-binding protein, calmodulin (CaM), interacts with SNARE to activate CaMKII, which is essential for actin polymerization and phagolysosome formation [43–45]. However, *M.tb* can evade Ca^2+^-induced phagolysosomal fusion and bacterial killing by reducing CaM and CaMKII levels and inhibiting sphingosine kinase [23, 46]. In this study, overexpression of bCoronin-1A resulted in a decrease in intracellular Ca^2+^ levels and down-regulated the expression of CaMKII and its activated forms. We hypothesized that bCoronin-1A inhibited phagolysosomal fusion through inhibition of the Ca^2+^-CaM-CaMKII pathway, on the contrary to prior reports that suggested it inhibited fusion by activating the Ca^2+^/calcineurin pathway [17]. This controversy about the role of Ca^2+^ in *M.tb* infection could have arisen because Ca^2+^ is a ubiquitous second messenger, which can activate a variety of downstream signaling molecules to produce different effects [47]. *M.tb* can exploit changes in physiological Ca^2+^ flux for their own survival, such as by preventing lysosomal clearance caused by elevated Ca^2+^ [22]. In this study, we used RAW264.7 and EBL cells overexpressing bCoronin-1A, and differences in cell models can have a definite effect on Ca^2+^-activated signaling pathways, which are influenced by the spatial and temporal organization of Ca^2+^ flux [47]. The intracellular calcium concentration is in dynamic equilibrium, and different stimuli can induce transient fluctuations of intracellular Ca^2+^ [48]. In this study, fluo-4AM was used to detect intracellular Ca^2+^ levels at 4 h after H37Ra infection, in contrast to a previous study that utilized Rhod-2a.m immediately after BCG infection. Differences in timing and reagents could influence the data. Furthermore, different strains show different extents of host invasion, and pathogenesis. The virulent strain H37Rv, the avirulent strain H37Ra and attenuated BCG have been widely investigated to identify virulence determinants based on mRNA, protein and macrophage response levels [49, 50].

We suspected that bCoronin-1A might interact with CaM because of earlier studies showing that proteins containing a WD repeat domain could bind to CaM in the presence of calcium [51]. However, we did not detect any interaction between the two (data not shown). It is still unclear how the WD repeat domain of bCoronin-1A inhibits the CaM-CaMKII pathway by regulating Ca^2+^, In addition, the reason for the upregulation of bCoronin-1A caused by *M.tb* infection needs to be further explored. Identifying the key regulatory regions could provide the basis for future modifications using gene editing technology to improve bovine tuberculosis resistance.

**Figure 6:**
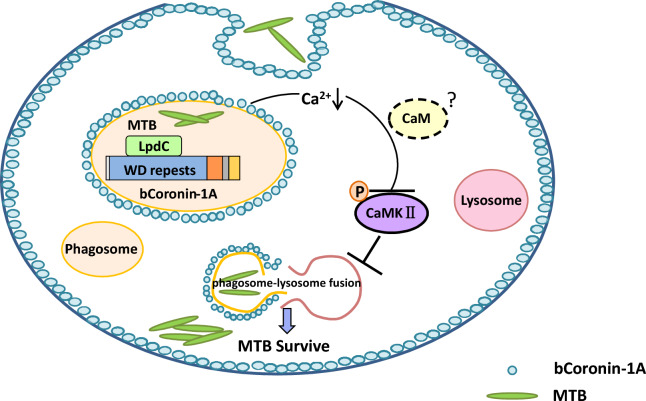
**Diagram showing bCoronin-1A inhibition of** ***M.tb*-containing phagolysosomes after fusion**. The WD repeat domain of bCoronin-1A interacts with the LpdC of *M.tb*, is retained on the phagosome, and inhibits the Ca^2+^-CaM-CaMKII pathway by reducing intracellular Ca^2+^ levels to assist *M.tb* in avoiding lysosomal clearance.

Considering the results as a whole, our study first proved that bCoronin-1A interacted with LpdC of *M.tb* via a WD repeat domain. This resulted in its being retained on phagosomes and preventing lysosomal trafficking by decreasing intracellular Ca^2+^, suggesting that bCoronin-1A may be a potential target for enhancing bovine resistance to *M.tb* (Figure 6).

## Supplementary Information

**Additional file 1. The bCoronin-1A protein is conserved and does not affect the adhesion of** ***M.tb*** **to macrophages**. (A) Phylogenetic tree of multi-species Coronin-1A. (B) Comparison of 3D structures of *Homo sapiens*, *Mus musculus* and *Bos taurus* Coronin-1A. (C) Macrophages overexpressing bCoronin-1A were infected with *M.tb* for 1 h, non-adherent bacteria were removed, and infection was continued until 4 h at 37 ℃. H37Ra labeled with FITC (*green*) and nuclei stained with DAPI (*blue*). (D) Statistical analysis of the percentage of cells with *M.tb* adhesion. (E) The fluorescence intensity of FITC-labeled *M.tb* was detected by a microplate reader. Data were analyzed by *t*-test and presented as the mean ± SEM of three independent experiments. ^*ns*^*P* > 0.05.

**Additional file 2. The bCoronin-1A protein and its family members interact with LpdC of** ***M.tb***. (A, C and D). The interaction of the following proteins with LpdC in vitro was detected by BiFc assay: (A) bCoronin-1A and its truncated forms; (C) bCoronin-1A and its family members; (D) bCoronin-1A and its WD repeat domain truncated forms. (B) Comparison of the WD repeat domain of bCoronin-1A with that of its family members.

**Additional file 3. The WD repeat domain mediates a decrease of intracellular calcium and inhibits CaMKII activation in EBL cells**. (A) Flow cytometry was performed to detect intracellular calcium levels labeled with fluo-4AM after 4 h of infection with *M.tb* in cells overexpressing bCoronin-1A (loaded or not loaded with 1 μM ionomycin for 30 min in 37 ℃), ΔW, and control cells (MOI = 10). (B) Western blot analysis of CaMKII and phospho-CaMKII (Thr286) expression levels after 4 h of infection with *M.tb* in cells overexpressing bCoronin-1A, ΔW, and control cells (MOI = 10). *P < 0.05.

**Additional file 4. The coiled-coil domain mediates trimer formation of bCoronin-1A and inhibits phagolysosomal fusion**. (A-B) SDS-PAGE and native-PAGE were used to detect the molecular forms of bCoronin-1A, ΔW and ΔC proteins. (C) Immunofluorescence localization of *M.tb* after 4 h of infection in cells overexpressing bCoronin-1A, ΔC and control cells. (D) Statistical analysis of the percentage of *M.tb* co-localized with lysosomes in the above cells. Data were analyzed by *t*-test and presented as the mean ± SEM of three independent experiments. * P < 0.05, *** P < 0.001.

## Data Availability

The datasets supporting the conclusions of this article are included within the article and its additional files.
