# Supplementary figures and images for: The tryptophan-aspartate (WD) repeat domain of bovine Coronin-1A promotes mycobacterial survival by inhibiting calcium signaling-mediated phagosome-lysosome fusion
Source: Vet Res. 2025 Feb 7;56:33. doi: 10.1186/s13567-025-01471-6 (PMC11806767; doi:10.1186/s13567-025-01471-6)

A

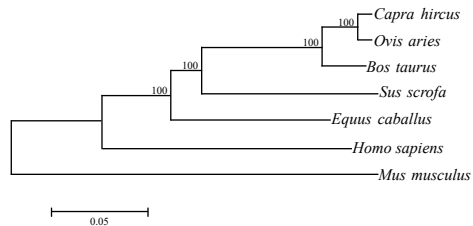

B

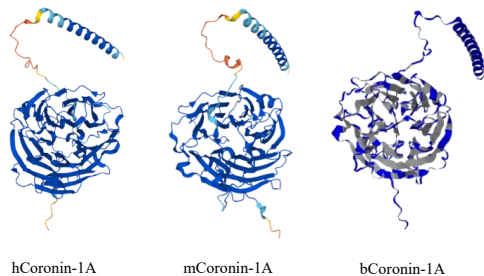

C

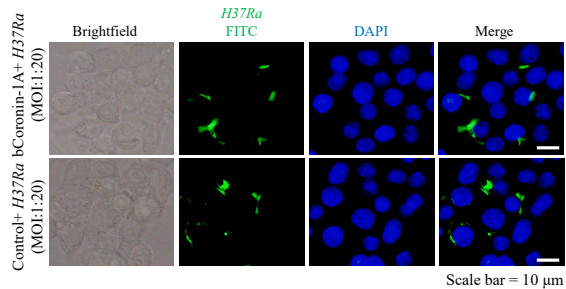

D

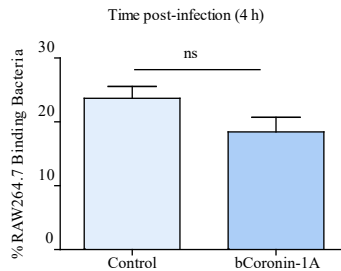

E

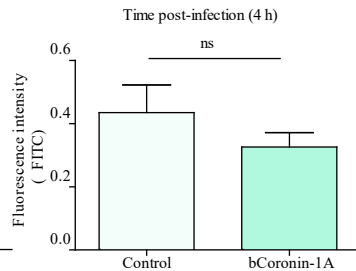

Supplement: Supplementary file 1 — Additional file 1. The bCoronin-1A protein is conserved and does not affect the adhesion of M.tb to macrophages. (A) Phylogenetic tree of multi-species Coronin-1A. (B) Comparison of 3D structures of Homo sapiens, Mus musculus and Bos taurus Coronin-1A. (C) Macrophages overexpressing bCoronin-1A were infected with M.tb for 1 h, non-adherent bacteria were removed, and infection was continued until 4 h at 37 ℃. H37Ra labeled with FITC (green) and nuclei stained with DAPI (blue). (D) Statistical analysis of the percentage of cells with M.tb adhesion. (E) The fluorescence intensity of FITC-labeled M.tb was detected by a microplate reader. Data were analyzed by t-test and presented as the mean ± SEM of three independent experiments. nsP > 0.05. [file 13567_2025_1471_MOESM1_ESM.pdf]

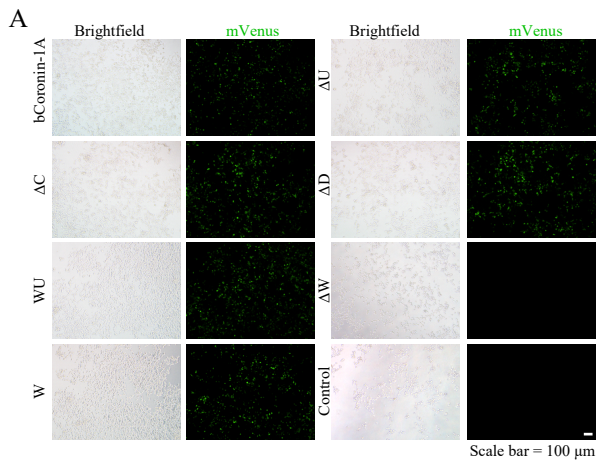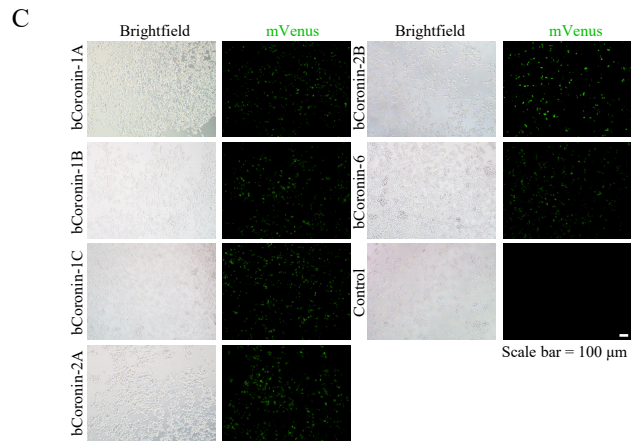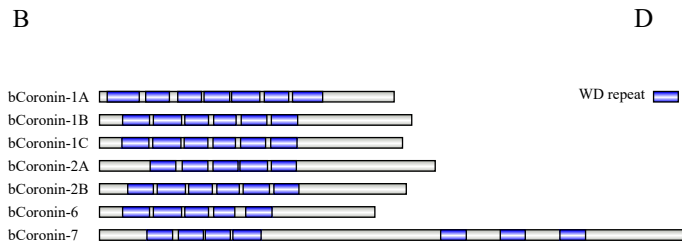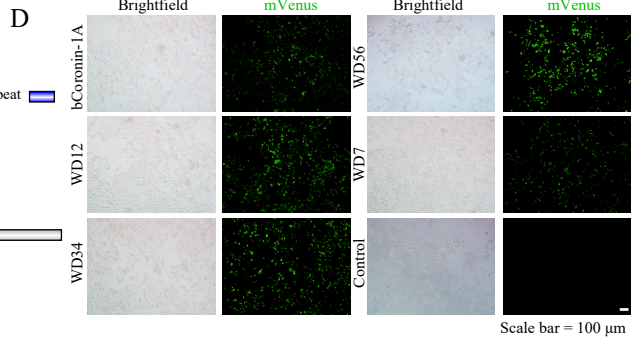

Supplement: Supplementary file 2 — Additional file 2. The bCoronin-1A protein and its family members interact with LpdC of M.tb. (A, C and D). The interaction of the following proteins with LpdC in vitro was detected by BiFc assay: (A) bCoronin-1A and its truncated forms; (C) bCoronin-1A and its family members; (D) bCoronin-1A and its WD repeat domain truncated forms. (B) Comparison of the WD repeat domain of bCoronin-1A with that of its family members. [file 13567_2025_1471_MOESM2_ESM.pdf]

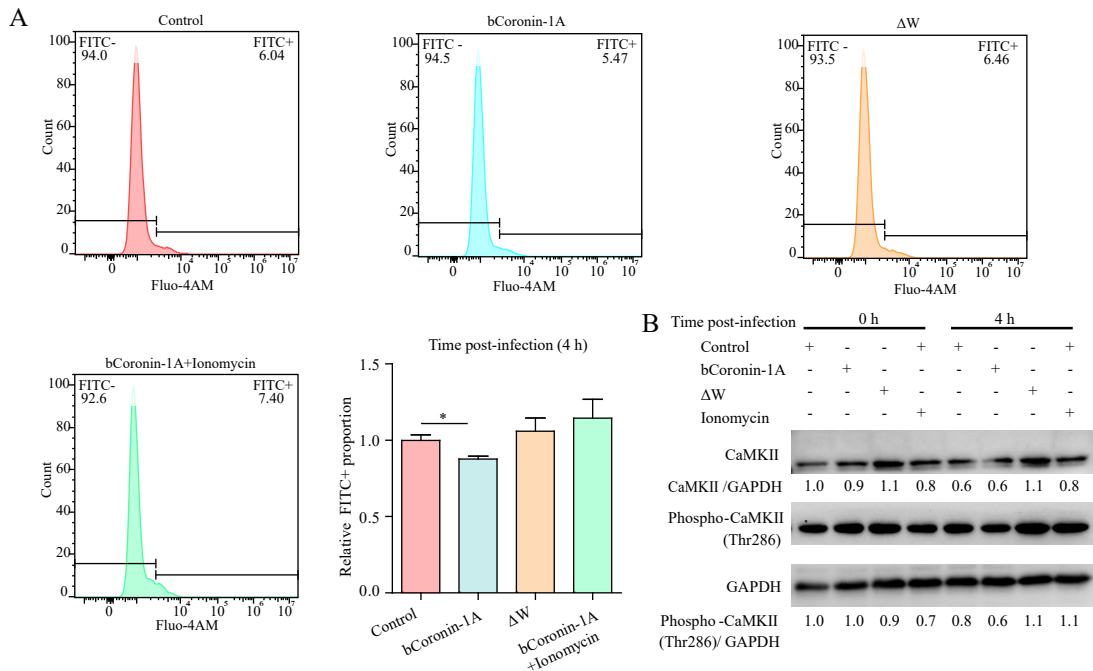

Supplement: Supplementary file 3 — Additional file 3. The WD repeat domain mediates a decrease of intracellular calcium and inhibits CaMKII activation in EBL cells. (A) Flow cytometry was performed to detect intracellular calcium levels labeled with fluo-4AM after 4 h of infection with M.tb in cells overexpressing bCoronin-1A (loaded or not loaded with 1 μM ionomycin for 30 min in 37 ℃), ΔW, and control cells (MOI = 10). (B) Western blot analysis of CaMKII and phospho-CaMKII (Thr286) expression levels after 4 h of infection with M.tb in cells overexpressing bCoronin-1A, ΔW, and control cells (MOI = 10). *P < 0.05. [file 13567_2025_1471_MOESM3_ESM.pdf]

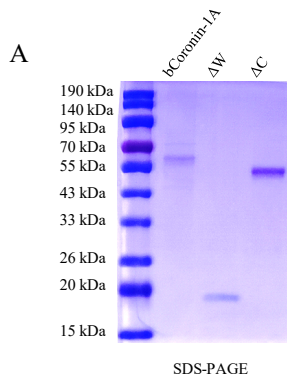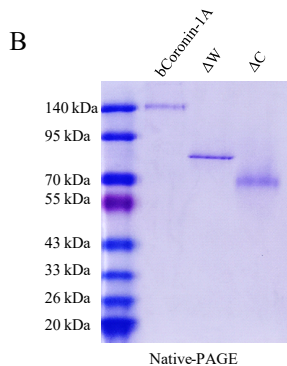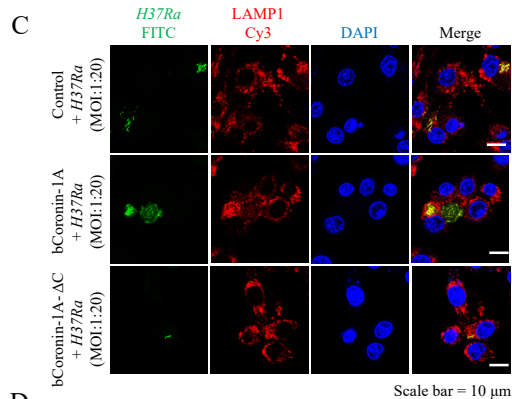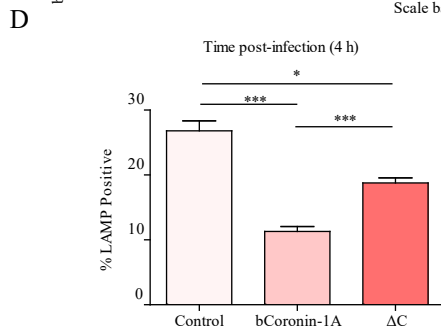

Supplement: Supplementary file 4 — Additional file 4. The coiled-coil domain mediates trimer formation of bCoronin-1A and inhibits phagolysosomal fusion. (A-B) SDS-PAGE and native-PAGE were used to detect the molecular forms of bCoronin-1A, ΔW and ΔC proteins. (C) Immunofluorescence localization of M.tb after 4 h of infection in cells overexpressing bCoronin-1A, ΔC and control cells. (D) Statistical analysis of the percentage of M.tb co-localized with lysosomes in the above cells. Data were analyzed by t-test and presented as the mean ± SEM of three independent experiments. * P < 0.05, *** P < 0.001. [file 13567_2025_1471_MOESM4_ESM.pdf]
